# Theory-Based Antecedents of Stopping Texting While Driving Among College Students for Injury Prevention: A Cross-Sectional Study

**DOI:** 10.3390/ijerph22121847

**Published:** 2025-12-10

**Authors:** Manoj Sharma, Sidath Kapukotuwa, Sharmistha Roy, Mahsa Pashaeimeykola, Asma Awan

**Affiliations:** 1 Department of Social and Behavioral Health, School of Public Health, University of Nevada, Las Vegas, NV 89154, USA; manoj.sharma@unlv.edu (M.S.); sidath.k@unlv.edu (S.K.); roys5@unlv.nevada.edu (S.R.); mahsa.pashaeimeykola@unlv.edu (M.P.); 2 Department of Internal Medicine, Kirk Kerkorian School of Medicine at UNLV, Las Vegas, NV 89102, USA; 3 Office of Research, Kirk Kerkorian School of Medicine at UNLV, Las Vegas, NV 89102, USA

**Keywords:** texting while driving, distracted driving, college students, health behavior, Multi-Theory Model (MTM), road safety

## Abstract

**Highlights:**

**Public health relevance—How does this work relate to a public health issue?**

- Texting while driving is a major preventable cause of road traffic injuries and deaths; in the U.S. alone, distracted driving killed 3142 people in 2022, making it a leading behavioral risk factor comparable to alcohol-impaired driving in younger age groups.
- It meets classic public health criteria for priority action: high burden of disease (morbidity and mortality), clear preventability through behavior change and policy, and large equity implications, as young drivers (15–29 years), a vulnerable population, are disproportionately affected.

**Public health significance—Why is this work of significance to public health?**

- Successful interventions could prevent thousands of deaths and hundreds of thousands of injuries annually worldwide; the WHO estimates that eliminating phone use while driving could reduce road traffic deaths by up to 10–15% in high-income countries.
- It represents a high-impact opportunity for primary prevention using multi-level approaches (education, enforcement, engineering, and environmental design), serving as a model for addressing other modern behavioral risks (e.g., social media addiction, sedentary behavior).

**Public health implications—What are the key implications or messages for practitioners, policy makers and/or researchers in public health?**

- Policy makers should prioritize comprehensive legislation (hands-free laws, graduated licensing restrictions for novices, and primary enforcement) combined with high-visibility enforcement campaigns; evidence shows states with strong texting bans and enforcement see 8–16% reductions in crashes.
- Practitioners and researchers should shift from awareness-only education (low effectiveness) toward evidence-based strategies: parental management apps and monitoring for teens, workplace fleet policies, integration of phone-blocking technology in vehicles, and rigorous evaluation of emerging driver-monitoring systems using public health trial designs.

**Abstract:**

Texting while driving (TWD) is a leading cause of distracted driving-related crashes, especially among college students. This study applied the Multi-Theory Model (MTM) of health behavior change to predict initiation and sustenance of refraining from TWD among university students. A cross-sectional survey was conducted among 164 students from a Southwestern U.S. public university using a 49-item validated MTM-based questionnaire. Structural equation modeling and hierarchical multiple regression analyses were employed to assess reliability, construct validity, and predictors of behavioral initiation and sustenance. Cronbach’s alpha coefficients ranged from 0.71 to 0.93, indicating strong reliability. The MTM demonstrated good fit (CFI = 0.950, RMSEA = 0.057 for initiation; CFI = 0.992, RMSEA = 0.039 for sustenance). Behavioral confidence (β = 0.30, *p* < 0.001) significantly predicted initiation, explaining 51.5% of the variance, while emotional transformation (β = 0.41, *p* < 0.001) and practice for change (β = 0.27, *p* = 0.0105) predicted sustenance, accounting for 61.5% of the variance. The MTM effectively explained both initiation and sustenance of refraining from TWD among college students. Interventions aimed specifically at reducing texting while driving should prioritize strengthening behavioral confidence for initiating change and supporting emotional transformation and practice-for-change strategies to sustain long-term abstinence from TWD. MTM-based approaches hold strong potential for designing theory-driven, culturally relevant distracted driving prevention programs.

## 1. Introduction

Texting while driving (TWD) is one of the most hazardous and prevalent forms of distracted driving, posing a significant threat to road safety worldwide [1,2]. TWD is defined as the act of composing, sending, or reading text messages, emails, or interacting with mobile applications while operating a motor vehicle [3]. Unlike other distractions, TWD uniquely combines visual (eyes off the road), manual (hands off the wheel), and cognitive (mind off driving) distractions, each of which independently impairs driving performance [4]. Simulation studies have demonstrated that drivers who text spend up to 400% more time looking away from the road, have significantly delayed hazard detection, and are up to five times more likely to be involved in a crash compared to undistracted drivers [5]. Moreover, reaction times while texting are worse than those of intoxicated drivers [6].

Globally, motor vehicle crashes account for approximately 1.35 million deaths annually and represent the leading cause of mortality among individuals under 30 years of age [7]. National estimates of texting while driving vary widely across studies, ranging from 14% to 31%, depending on sample characteristics, measurement methods, and state-level enforcement differences [7]. In the United States alone, distracted driving resulted in 3275 fatalities and approximately 324,819 injuries in 2023, with cellphone use, including texting, identified as a significant contributing factor [8]. Among drivers aged 15 to 20 involved in fatal crashes, 7% were reported to be distracted, representing the highest proportion of distracted drivers across age groups [8].

From a public health perspective, TWD is a critical and modifiable risk behavior with severe consequences that extend beyond immediate crash risks [5]. Short-term effects include impaired attention, delayed reaction times, erratic lane maintenance, and increased crash severity [1]. Long-term consequences may include chronic stress, emotional trauma, legal penalties, increased insurance premiums, and substantial societal costs associated with emergency medical care and lost productivity [9,10].

College students represent a particularly high-risk group for TWD due to their frequent mobile phone use, a strong culture of multitasking, and both a perceived sense of invincibility and an underestimation of the danger associated with the behavior [11,12]. Multiple studies highlight alarmingly high prevalence rates, such as [13], which reported that 91% of college students admitted to TWD, while [14] found that 75% of undergraduates had sent texts or emails while driving, and 82% had read them. Although younger drivers often report a belief that they can multitask safely, research shows that while they may perform texting-while-driving tasks better than older drivers, they are nevertheless not safe when doing so [15]. Despite widespread awareness of its dangers, college students continue to engage in TWD due to factors such as perceived social norms favoring constant connectivity, academic pressures, stress, and a false belief in their ability to multitask safely [14,16]. Existing interventions, including public service announcements, educational campaigns, and legal bans on TWD, have shown limited success in changing behavior among this group. Enforcement of these laws is inconsistent, and although awareness has increased, these measures often fail to produce sustained behavioral change [17].

In addition to individual-level behavioral determinants, broader contextual influences also shape risky driving tendencies. The Contextual Mediated Model of Traffic Accident Involvement distinguishes between distal factors, such as personality traits, attitudes, sociodemographic characteristics, cultural norms, and policy or regulatory environments and proximal factors, which include behaviors that occur directly behind the wheel. Within this framework, texting while driving represents a proximal behavioral factor that may be shaped by distal contextual conditions, including regional traffic norms and patterns of mobile phone use. Prior research on distracted driving and problematic mobile-phone use also highlights the importance of accounting for these multilevel influences when examining texting while driving behaviors [12,18,19,20]. Integrating this perspective situates the MTM constructs within a broader contextual environment that contributes to risky driving behavior.

To understand and address TWD more effectively, several behavioral theories have been applied, including the Theory of Planned Behavior (TPB), Health Belief Model (HBM), and Prototype Willingness Model (PWM) [2,14]. While these models have provided valuable insights into social norms, attitudes, and perceived behavioral control, they primarily focused on predicting intentions rather than supporting the maintenance of behavior change over time [21,22]. To address this limitation, the present study adopts the Multi-Theory Model (MTM) of health behavior change, a contemporary, fourth-generation framework specifically designed to predict both initiation and sustenance of health behaviors [23]. While traditional approaches such as testimonials and personal stories can be effective in some settings, theoretical models like MTM provide structured, mechanism-based insights that may enhance the design and targeting of TWD prevention interventions. Moreover, prior behavioral models often demonstrate relatively low explanatory power, as reflected by their lower adjusted R^2^ values, whereas newer models account for a greater proportion of variance in TWD-related behaviors [24].

Historically, the evolution of health behavior models has progressed through several generations. First-generation models emphasized knowledge dissemination, while second-generation models focused on skill acquisition. Third-generation models targeted behavior acquisition, aiming to instill specific health behaviors. In contrast, the novice fourth-generation models represent a significant advancement by integrating constructs from multiple evidence-based theories to facilitate actual behavior change. These models emphasize the development of precise, tailored interventions that address both the initiation and maintenance of behavior [23]. A prominent example is the Multi-Theory Model (MTM) of health behavior change, which incorporates cognitive, conative, volitional, and environmental components to support sustainable behavioral transformation [25]. The MTM has demonstrated effectiveness in predicting and promoting health behavior change across several domains, including smoking cessation, safer sexual practices, physical activity, portion control, and avoidance of risky alcohol use. Studies have shown that MTM-based interventions often outperform traditional educational approaches by targeting specific cognitive and emotional mechanisms of change rather than relying on general awareness messaging [23,25,26]. Thus, the MTM offers both a practical intervention framework and a theory-driven approach to structuring behavioral modification efforts.

Given this theoretical progression, the adoption of fourth-generation models such as the MTM is essential for understanding the determinants of TWD and for designing effective, age-appropriate, and culturally sensitive interventions for college students.

The MTM is a novel fourth-generation theoretical model that facilitates both the adoption and long-term maintenance of new behaviors. It divides the behavior change process into two distinct but interrelated phases: initiation and sustenance [23]. The initiation phase involves participatory dialogue, behavioral confidence, and adjustments to the physical environment. Participatory dialogue refers to the process of weighing up the advantages and disadvantages of adopting a new behavior. Positive dialogue increases the likelihood of initiating change when benefits outweigh drawbacks [23]. In the context of TWD, emphasizing safety and legal benefits can motivate individuals to quit. Behavioral confidence, similar to self-efficacy, refers to one’s confidence in one’s ability to perform a behavior change despite challenges. For college students, this means resisting the urge to check or respond to messages while driving. Changes in the physical environment involve modifying surroundings to support behavior change, such as using apps to block notifications, storing phones out of reach, or setting automatic replies.

The sustenance phase encompasses emotional transformation, practice for change, and adjustments to the social environment. Emotional transformation refers to converting emotions into goals that support the ongoing practice of the new behavior. For example, turning fear of accidents into a personal commitment to never text while driving can reinforce long-term adherence [23]. Practice for change involves developing cognitive and behavioral strategies, such as self-reflection and problem-solving, to handle situations that might prompt relapse. Changes in the social environment include leveraging support from friends, family, and peers to reinforce and sustain behavior change. In college settings, peer pledges and supportive networks can play a crucial role in promoting consistent avoidance of TWD.

The MTM’s unique and dual focus on initiation and sustenance makes it particularly well-suited for addressing TWD among college students, a group heavily influenced by social norms and accustomed to constant connectivity. The theoretical constructs demonstrate flexibility and have been effectively applied to a wide range of health behaviors, particularly among college student populations. This adaptability highlights their relevance and utility in diverse behavioral contexts within this demographic group [27,28,29,30,31,32]. By targeting both the initial decision to stop and the maintenance of this safer behavior, the MTM addresses key gaps left by previous theoretical models. This study is the first known application of the MTM to TWD cessation among college students. Therefore, our study aims to utilize the MTM to explain the antecedents of stopping TWD behavior while also predicting the maintenance of this behavior to prevent injury among college students. In addition, the study will help design culturally appropriate, theory-driven interventions that not only motivate college students to refrain from TWD but also help them maintain this change over time. It will also address one of the most pressing public health issues of our time by promoting safer driving practices and supporting ongoing efforts in injury prevention, thereby expanding evidence-based knowledge and informing effective policy shifts.

## 2. Materials and Methods

### 2.1. Study Design, Sample, and Participants

This study adopted a cross-sectional design to examine determinants of refraining from texting while driving among college students using the Multi-Theory Model (MTM) of health behavior change. The sample size was calculated using G*Power 3.1.9.7 software (Heinrich-Heine-Universität Düsseldorf) [33], assuming an alpha value of 0.05, power of 0.80, a medium effect size of 0.15, and nine predictors (three MTM constructs in each model and six covariates). The computed sample requirement was 114, which was increased by 15 percent to account for possible incomplete responses. The user data were gathered from April 2025 to June 2025 from public university students in the Southwestern region of the United States. The potential participants must be aged 18 years or more, English speakers, and undergraduate or graduate students. Voluntary participation was allowed, but only subjects with informed consent were included for the final analysis.

In addition to the a priori power analysis conducted using G*Power for the hierarchical regression, we also conducted SEM-specific sample size estimation to ensure adequate statistical power for the structural equation modeling analyses. SEM sensitivity was evaluated using the SEM sample size calculator (Daniel Soper, Calculator #89). For the initiation model, we specified an anticipated effect size of 0.30, desired statistical power of 0.80, five latent variables, twenty-four observed variables, and an alpha level of 0.05. The recommended minimum sample size was 150. For the sustenance model (four latent variables and thirteen observed variables), the recommended sample size was 166.

### 2.2. Ethical Considerations

Ethical approval for carrying out this research was received from the University of Nevada, Las Vegas, Institutional Review Board on protocol number UNLV-2025-120 on 16 April 2025. All the procedures conformed to institutional and federal ethical guidelines. Participants were aware of the reason for the study, the anticipated benefits and risks, and that they had the right to withdraw at any time without penalty. Informed consent was collected electronically using a Qualtrics-based consent script; participants only moved on to the survey if they clicked “Agree.” No identifying data, including names, student ID numbers, or email addresses, was collected. All responses were saved in encrypted files that only members of the research team could access, ensuring confidentiality and data security.

### 2.3. Recruitment and Data Collection

Participant recruitment was carried out through direct university email announcements distributed with approval from the registrar’s office. The sample included a mix of commuting and non-commuting students; however, we did not compare these proportions to the university’s overall distribution, which may affect generalizability. The invitation email described the purpose of the research and provided a secure, anonymous survey link hosted on the Qualtrics platform (Provo, UT, USA). To maintain data integrity, several quality-control features were enabled within Qualtrics. The “Prevent multiple submissions” function was activated to ensure that respondents could submit only one survey. Bot detection and RelevantID verification tools were used to identify and remove fraudulent or duplicate entries. Incomplete or invalid responses were excluded during the data-cleaning process. The survey required approximately 15 to 18 min to complete.

A total of 250 valid survey responses were obtained. Among these, 164 participants reported engaging in texting while driving. Consistent with the analytic strategy, only the responses of these 164 individuals were included in the hierarchical regression analyses. However, all valid survey responses (*n* = 250) were included in the SEM analyses, as the MTM constructs were assessed for the full sample and the SEM models were not restricted to participants who engaged in texting while driving.

### 2.4. Survey Instrument

A forty-nine-item questionnaire was developed based on the constructs of the Multi-Theory Model (MTM) of health behavior change (File S1: Survey Questionnaire). The instrument was adapted from previously validated MTM tools developed by Sharma [34,35,36,37,38] and modified to address the specific target behavior of stopping texting while driving. A rigorous two-stage expert validation process was used to establish face and content validity. The expert panel consisted of five members: two specialists in behavioral theory, health education, and instrument development; two experts in young adult health promotion; and one expert in health administration and survey design. Two panel members also had direct experience working with college students and survey research. Each expert evaluated the items for clarity, relevance, and theoretical alignment. No items were removed, but several were revised slightly to improve readability and comprehension. The final version of the questionnaire demonstrated strong readability with a Flesch Reading Ease score of 79.7 and a Flesch–Kincaid Grade Level of 5.4, indicating that college students easily understood it.

The survey began with three screening items to establish eligibility, asking if participants owned a cell phone, drove a vehicle, and had texted while driving in the past thirty days. The participants who responded “no” to questions one and two were asked to end the survey. The rest assessed MTM’s core constructs, which were framed under initiation and sustenance factors. Participatory dialogue representing the initiation construct was assessed with twelve items that were classified into two subdomains: perceived benefits (Items 5–10) and perceived disadvantages (Items 11–16). The items were all scored on a five-point Likert-type scale from 0 (never) to 4 (very often).

The participatory dialogue score was obtained by subtracting the disadvantages score from the advantages score, producing a possible range from −24 to +24, where higher positive values indicated a greater likelihood of initiating behavior change. Behavioral confidence, another initiation construct, was assessed using six items (Items 17–22) rated on a five-point scale from 0 (not at all sure) to 4 (completely sure), yielding a total possible score ranging from 0 to 24. Changes in the physical environment were measured using three items (Items 23–25) on the same five-point confidence scale, with a total range from 0 to 12.

The sustenance constructs included emotional transformation, practice for change, and changes in the social environment. Emotional transformation was measured using three items (Items 26–28) that assessed the participant’s ability to channel emotions and maintain motivation to stop texting while driving, with total possible scores ranging from 0 to 12. Practice for change was evaluated using three items (Items 29–31) that focused on the ability to self-monitor and adapt to overcome barriers, with a total possible score of 0 to 12. Changes in the social environment were measured using four items (Items 32–35) assessing the degree of social support received from family members, friends, social media, and passengers, producing a total possible range of 0 to 16. The intent to initiate and sustain the behavior was assessed using six items (Items 36–41), which employed a five-point Likert scale ranging from 0 (not at all likely) to 4 (completely likely). The intent-to-initiate subscale (Items 36–38) and the intent-to-sustain subscale (Items 39–41) each had possible score ranges from 0 to 12, with higher scores representing stronger behavioral intentions.

Finally, the survey also contained seven demographic questions ranging from gender and age to race or ethnicity, class status, GPA, residency status, and hours worked per week. Internal consistency was calculated using Cronbach’s alpha, and the cut-off was set at 0.70. The subscales with higher scores indicated a greater readiness to initiate and sustain the behavior of not texting while driving.

### 2.5. Statistical Analyses 

All statistical analyses were performed using SAS software, version 9.4 (SAS Institute Inc., Cary, NC, USA), and R Statistical Software, version 4.5.1 (R Core Team, 2021). Descriptive statistics were presented as means and standard deviations for continuous variables, and as frequencies and percentages for categorical variables.

Internal consistency was evaluated using Cronbach’s alpha coefficients for both the overall scale and individual subscales representing specific theoretical constructs. An alpha value of 0.70 or higher was considered the minimum acceptable threshold, indicating adequate reliability of the measures.

To further examine reliability, unidimensionality, and construct validity, structural equation modeling (SEM) was conducted in R, treating each scale as a latent construct. Construct validity was assessed by analyzing correlations among latent factors within the measurement model. Model fit was evaluated using multiple fit indices, including the Comparative Fit Index (CFI) and Tucker–Lewis Index (TLI), with values greater than 0.90 indicating acceptable fit, and the Root Mean Square Error of Approximation (RMSEA) and Standardized Root Mean Square Residual (SRMR), with values below 0.08 denoting good model fit.

The study focused on two primary outcomes: the intention to stop texting while driving (initiation) and the intention to maintain this behavior change (sustenance). These outcomes were evaluated using constructs derived from the Multi-Theory Model (MTM) of health behavior change. Independent variables for initiation included participatory dialogue, behavioral confidence, and changes in the physical environment. At the same time, sustenance was assessed through emotional transformation, practice for change, and changes in the social environment. Covariates incorporated into the analysis were age, gender, race/ethnicity, class level, GPA, and self-reported problematic texting behavior. Because some participants did not report their age, a separate model without including age as a covariate was tested. This model was ultimately selected as the final model for both initiation and sustenance.

Structural Equation Modeling (SEM) was employed to assess the construct validity of the MTM framework and to evaluate model fit for both initiation and sustenance. Model adequacy was determined using established fit indices, including the RMSEA, SRMR, CFI, and TLI. Hierarchical multiple regression analyses were conducted to identify predictors of the intention to initiate and sustain the behavior change. In the initiation model, demographic and behavioral covariates were entered first (Base model), followed by participatory dialogue, behavioral confidence, and changes in the physical environment (Final model). A similar procedure was applied to the sustenance model, in which emotional transformation, practice for change, and changes in the social environment were applied to the base model. This approach allowed for examination of the contribution of MTM constructs while controlling covariates. Assumptions of independence, linearity, normality, and homoscedasticity were tested and confirmed using the Durbin–Watson statistic, partial regression plots, the Shapiro–Wilk test, and the White test, respectively. Multicollinearity was evaluated through the Variance Inflation Factor (VIF). All statistical tests were two-tailed, with a significance level set at 0.05.

## 3. Results

### 3.1. Sample Characteristics

The study included 164 participants. However, only 163 participants reported their Gender, Race and Ethnicity and GPA. Also, only 127 participants reported their age. Furthermore, only 107 participants reported their working hours. The average age of respondents (*n* = 127) was 29.9 years (SD = 10.8), and participants worked an average of 31.5 h per week (SD = 14.1; *n* = 107). Females represented most of the sample (72.4%, *n* = 118), while males accounted for 27.6% (*n* = 45). Racial and ethnic composition showed that 37.4% (*n* = 61) identified as White or Caucasian American, 28.2% (*n* = 46) as Hispanic American, 17.8% (*n* = 29) as Asian American, and 10.4% (*n* = 17) as Black or African American. A smaller proportion identified as American Indian (0.61%, *n* = 1) or other backgrounds (5.5%, *n* = 9). Over half of participants (50.6%, *n* = 83) were enrolled in graduate programs, followed by juniors (21.3%, *n* = 35), seniors (13.4%, *n* = 22), sophomores (9.2%, *n* = 15), and freshmen (5.5%, *n* = 9). Grade point averages indicated that most students (68.1%, *n* = 111) earned between 3.50 and 4.00, with 23.3% (*n* = 38) between 3.00–3.49, 7.4% (*n* = 12) between 2.50–2.99, and 1.2% (*n* = 2) between 2.00–2.49. Employment was common, as 84.3% (*n* = 107) were currently working, while 15.8% (*n* = 20) were not. Nearly all participants (95.7%, *n* = 157) lived off campus, with only a few (4.3%, *n* = 7) residing on campus. Most participants (71.3%, *n* = 117) described themselves as non-problematic texters. When asked about texting frequency during the previous week, 39.6% (*n* = 65) reported texting “sometimes,” 36.6% (*n* = 60) “almost never,” 16.5% (*n* = 27) “fairly often,” 6.1% (*n* = 10) “often,” and 1.2% (*n* = 2) “never” (Table 1).

### 3.2. Internal Consistency and Construct Validity

Table 2 summarizes the internal consistency estimates for the initiation and sustenance constructs examined in the study. Cronbach’s alpha coefficients for all scales and subscales exceeded or closely approached the recommended threshold of 0.70, suggesting satisfactory reliability. Specifically, Cronbach’s alpha values ranged from 0.71 for Perceived Disadvantage to 0.93 for the Overall Sustenance Scale and the Overall Scale, indicating strong internal consistency across the measures. These findings support the reliability of the instrument in evaluating the components of the Multi-Theory Model (MTM) related to behavior change initiation and sustenance.

**Figure 1:**
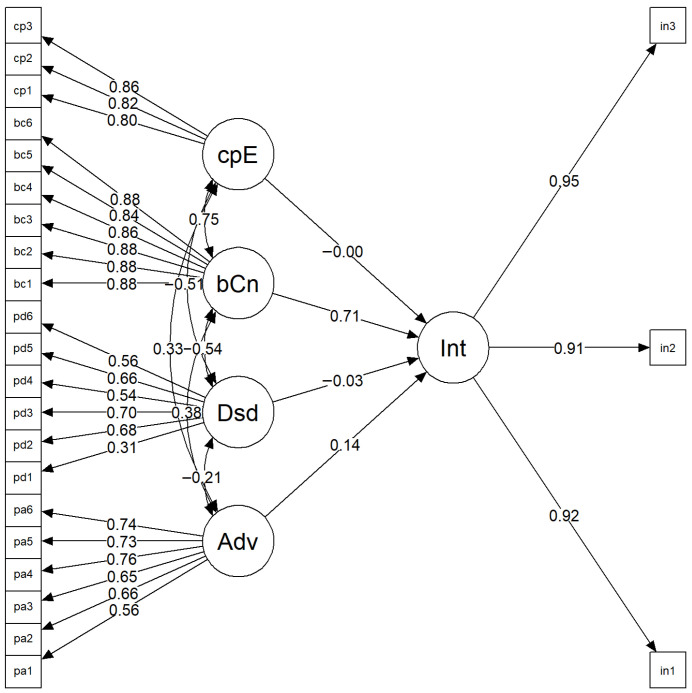
Path diagram of the structural equation model illustrating predictors of initiation. Note: Adv = Participatory Dialogue-Perceived Advantages; Dsd = Participatory Dialogue-Perceived Disadvantages; bCn = Behavioral Confidence; cpE = Changes in the Physical Environment; Int = Initiation; pa1–pa6 = items measuring perceived advantages; pd1–pd6 = items measuring perceived disadvantages; bc1–bc6 = items measuring behavioral confidence; cp1–cp3 = items measuring changes in the physical environment; in1–in3 = items measuring initiation behavior.

The structural equation modeling (SEM) analysis of the initiation model demonstrated an acceptable overall fit to the data (Figure 1). The model fit indices indicated a reasonably good fit, with a robust CFI of 0.950, TLI of 0.943, RMSEA of 0.057 (90% CI = 0.046–0.066), and SRMR of 0.051. These values fall within the recommended thresholds for adequate model fit, suggesting that the hypothesized model corresponds well with the observed data.

Standardized factor loadings for the latent constructs ranged from 0.31 to 0.95, indicating that the observed items contributed strongly to their respective latent variables. Regarding the structural paths, Initiation was significantly and positively predicted by Behavioral Confidence (β = 0.708, *p* < 0.001) and Perceived Advantage (β = 0.138, *p* = 0.027). In contrast, the effects of Perceived Disadvantage (β = −0.026, *p* = 0.625) and Changes in the Physical Environment (β = −0.001, *p* = 0.995) were not statistically significant.

Significant correlations were also found among several latent variables. Perceived Advantage was positively correlated with both Behavioral Confidence (r = 0.375, *p* < 0.001) and the Changes in Physical Environment (r = 0.333, *p* < 0.001), while Perceived Disadvantage was negatively related to both constructs (r = −0.545 and −0.508, respectively, *p* < 0.001).

The model accounted for approximately 61.6% of the variance in Initiation (R^2^ = 0.616), suggesting moderate to strong explanatory power. Overall, these findings underscore the central role of behavioral confidence and perceived advantages in driving initiation behavior, aligning with theoretical expectations that self-efficacy and perceived benefits serve as key motivators for action.

**Figure 2:**
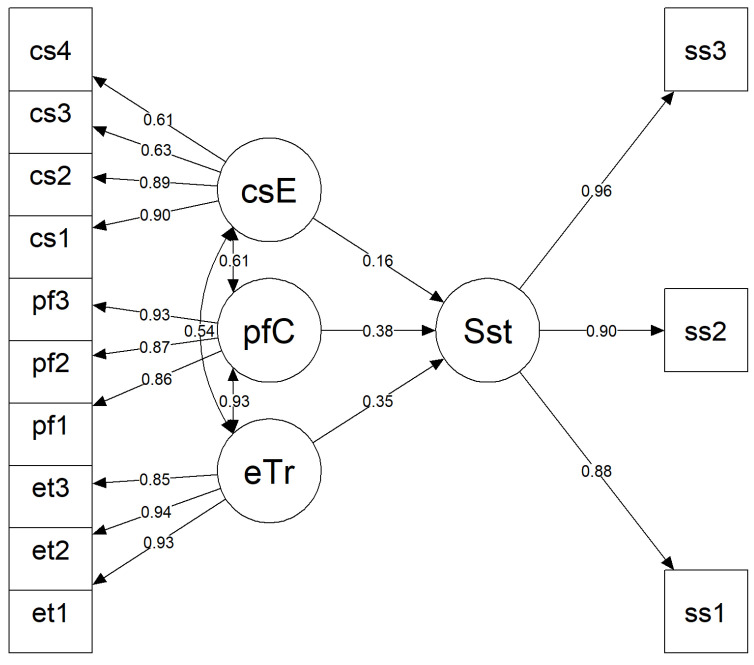
Path diagram of the structural equation model illustrating predictors of sustenance. Note: eTr = Emotional Transformation; pfC = Practice for Change; csE = Changes in the Social Environment; Sst = Sustenance; et1–et3 = items measuring emotional transformation; pf1–pf3 = items measuring practice for change; cs1–cs4 = items measuring changes in the social environment; ss1–ss3 = items measuring sustenance behavior.

The SEM analysis of the sustenance model demonstrated an excellent overall fit to the data (Figure 2). The model fit indices indicated a good fit, with a robust CFI of 0.992, TLI of 0.990, RMSEA of 0.039 (90% CI = 0.000–0.063), and SRMR of 0.031. These values fall within the recommended thresholds for excellent model fit, suggesting that the hypothesized model corresponds well with the observed data.

Standardized factor loadings for the latent constructs ranged from 0.61 to 0.96, indicating that the observed items contributed strongly to their respective latent variables. Regarding the structural paths, Sustenance was positively predicted by Emotional Transformation (β = 0.352, *p* = 0.082), Practice for Change (β = 0.380, *p* = 0.109), and Change in the Social Environment (β = 0.163, *p* = 0.085); however, these effects did not reach conventional levels of statistical significance.

Significant correlations were also found among several latent variables. Emotional Transformation was positively correlated with Practice for Change (r = 0.932, *p* < 0.001) and Change in the Social Environment (r = 0.539, *p* < 0.001), while Practice for Change was also positively associated with Change in the Social Environment (r = 0.612, *p* < 0.001).

The model accounted for approximately 68.2% of the variance in Sustenance (R^2^ = 0.682), indicating strong explanatory power. Overall, these findings suggest that while Emotional Transformation, Practice for Change, and Change in the Social Environment are positively related to sustenance, their effects were not statistically significant in this model. Nonetheless, the high correlations among predictors highlight their interrelated roles in supporting sustenance outcomes.

### 3.3. Characteristics of Study Variables and Inferential Statistics

Table 3 summarizes the descriptive statistics for the constructs of the multi-theory model of behavior change among the participants (*n* = 164). The results indicate moderate mean scores for both initiation (M = 7.6, SD = 3.3) and sustenance (M = 7.2, SD = 3.3), suggesting that participants demonstrated a comparable tendency to begin and maintain behavior change. Among the initiation sub-constructs, the mean score for perceived advantage (M = 18.8, SD = 4.3) was substantially higher than that for perceived disadvantage (M = 7.2, SD = 3.8), producing a positive overall score for participatory dialogue (M = 11.6, SD = 6.1). This indicates that participants generally recognized more benefits than barriers to behavior change. Behavioral confidence showed a relatively high mean score (M = 16.5, SD = 5.8), reflecting a strong sense of self-efficacy among respondents. Similarly, changes in the physical environment (M = 8.4, SD = 3.3) demonstrated moderate support from external surroundings conducive to change. For the sustenance phase, emotional transformation (M = 7.8, SD = 3.2), practice for change (M = 7.3, SD = 3.1), and changes in the social environment (M = 8.1, SD = 4.3) all reflect moderate engagement in emotional regulation, behavioral practice, and social support strategies necessary for maintaining positive behavior change.

Model diagnostics indicated that the specification of both regression models was appropriate. For the model predicting Initiation, the test of first and second moment specification was not significant (χ^2^(127) = 111.75, *p* = 0.8304), and the Durbin–Watson statistic suggested no evidence of autocorrelation (DW = 1.818; *p* = 0.8853). Similar results were observed for the model predicting Sustenance (χ^2^(128) = 118.15, *p* = 0.7224; DW = 1.779; *p* = 0.9283). However, the residuals did not meet the assumption of normality, as the Shapiro–Wilk tests were significant (Initiation: W = 0.965, *p* = 0.0004; Sustenance: W = 0.958, *p* < 0.0001). Despite the non-normality of residuals, the independence and correct model specification support the overall adequacy of the fitted models. Multicollinearity diagnostics showed no violations in both regression models. For the model predicting Initiation, all VIF values were between 1.12 and 2.14, indicating minimal collinearity. For the model predicting Sustenance, VIFs ranged from 1.14 to 3.99, remaining under conventional cutoffs for problematic multicollinearity. Overall, the predictor set demonstrates acceptable independence, supporting stable and reliable coefficient estimates.

The hierarchical multiple regression assessed whether MTM constructs improved the prediction of initiation beyond demographic and behavioral covariates (Table 4). The base model (covariates only, without age) was significant (F = 3.47, *p* < 0.0001; adjusted R^2^ = 0.1760). After adding MTM constructs, the final model remained highly significant (F = 11.12, *p* < 0.0001) with an adjusted R^2^ = 0.5151, indicating that 51.5% of the variance in initiation was explained. This represents a ΔR^2^ = 0.3391 increase over the base model, demonstrating substantial incremental explanatory power from the theoretical constructs.

The hierarchical multiple regression analysis was conducted to examine whether the addition of Multi-Theory Model (MTM) constructs improved the prediction of sustenance of the behavior beyond demographic and behavioral covariates (Table 5). The base model, including only covariates (without age), was statistically significant (F = 4.69, *p* < 0.0001; adjusted R^2^ = 0.2417). After adding the MTM constructs, the final model remained highly significant (F = 16.20, *p* < 0.0001) with an adjusted R^2^ = 0.6147, indicating that approximately 61.5% of the variance in sustenance was explained by the model. This represents a ΔR^2^ = 0.3730, reflecting a substantial improvement in predictive power after inclusion of the MTM constructs.

Among the theoretical constructs, Emotional Transformation (β = 0.41, *p* < 0.0001) and Practice for Change (β = 0.27, *p* = 0.0105) were statistically significant positive predictors of sustenance, suggesting that greater emotional commitment and consistent self-regulatory practices are key to maintaining the target behavior. The construct Changes in the Social Environment was not significant (β = 0.05, *p* = 0.3447).

Regarding covariates, sex (reference = female) was a significant negative predictor (β = −1.43, *p* = 0.0003), indicating that males reported lower sustenance scores than females. Participants who self-identified as problematic texters had significantly higher sustenance scores (β = 1.63, *p* < 0.0001), while sophomore students also demonstrated higher sustenance compared with graduate students (β = 1.46, *p* = 0.0245). Those reporting high-frequency texting behavior showed lower sustenance (β = −1.00, *p* = 0.0357), indicating potential behavioral interference. Race/ethnicity, GPA, and other class levels were not significant predictors in the final model.

Overall, these findings underscore Emotional Transformation and Practice for Change as core MTM constructs explaining sustained engagement in the behavior. The large increase in explained variance following inclusion of these constructs highlights their importance in long-term maintenance, supporting the utility of the MTM in predicting and reinforcing behavioral sustenance.

## 4. Discussion

The current study examined the predictors of initiation and sustenance for stopping texting while driving among college students using the Multi-Theory Model (MTM) of health behavior change. This is, to our knowledge, one of the first applications of the MTM to understand behavioral intentions surrounding distracted driving prevention in a university context. The findings demonstrated that MTM constructs significantly enhanced the explanatory power of both initiation and sustenance models beyond demographic predictors. Specifically, the MTM constructs along with some demographic covariates explained 51.5% of the variance in initiation and 61.5% of the variance in sustenance, highlighting the strong predictive utility of the MTM according to the standards in behavioral and social sciences [39].

For the initiation model, behavioral confidence was found to be a statistically significant predictor, as postulated in earlier research using the MTM for other risky driving and behavioral health problems [25,26]. These results suggest that students who are more behaviorally confident in their ability to do so are likely to plan to make this change. Further, the negative association with sex suggests that male students may demonstrate stronger initiation intentions compared to female students, aligning with previous findings that highlight gender differences in risk perception regarding distracted driving behaviors [25,26]. Self-identified problematic texting status and Black race/ethnicity also significantly contributed to the final model, underscoring the importance of self-awareness and the role of race. The MTM constructs of participatory dialogue and changes in the physical environment were not statistically significant, implying that perhaps, due to the target population’s youthfulness, they were already aware of the benefits and harms of not engaging in texting while driving, and they were not overtly concerned about making changes in the physical environment.

These findings are consistent with the broader MTM literature that positions behavioral confidence as a dominant construct across multiple health behaviors. The MTM has been described as a “fourth generation” theoretical framework that integrates elements from several behavioral theories and is particularly suitable for behaviors requiring both initiation and maintenance [40]. In applications such as handwashing [32], fruit and vegetable intake [35], and drowsy driving [25], behavioral confidence has repeatedly emerged as the strongest determinant of behavior change. The consistency of these findings across distinct behavioral domains reinforces the external validity of the MTM and supports its application to technology-related risk behaviors like texting while driving. However, while participatory dialogue and changes in physical environment were not significant in the current study, they have shown predictive value in other MTM studies, suggesting that contextual or cultural differences among college populations may moderate these relationships. Future studies should therefore explore how factors such as smartphone dependency, peer texting norms, or enforcement of state texting-while-driving laws might interact with MTM constructs to influence behavior change.

For the sustenance model, emotional transformation and practice for change were significant, mirroring patterns seen in prior MTM studies involving habitual behaviors such as drowsy driving, physical activity, and substance-use prevention [24,25,28]. These findings underscore the importance of maintaining emotional self-regulation and converting feelings into goals in sustaining a behavioral change that requires consistent self-monitoring. Additionally, emotional transformation highlights the need for interventions that help students channel emotional impulses, such as anxiety or urgency to respond to messages, toward safer communication behaviors. Furthermore, males reported significantly lower sustenance scores than females; those who self-identified as problematic texters had significantly higher sustenance scores; and sophomore students demonstrated higher sustenance scores compared with graduate students. This could perhaps be explained by more risk-taking by males and greater awareness among problematic texters and sophomores to change their behavior. The MTM construct of changes in the social environment was not significant, implying that texting while driving is more of a personal and situational phenomenon, so family members and friends may have a limited role in influencing its maintenance.

The results provide empirical support for the theoretical adequacy of the MTM in predicting technology-related behavioral risks. The increase in explained variance from demographic-only to MTM-enhanced models underscores that psychosocial constructs are stronger predictors of behavioral intention than demographic characteristics alone. The non-significance of constructs in initiation, such as participatory dialogue and changes in the physical environment, may reflect the contextual nature of texting while driving, where the behavior occurs in transient, mobile environments with limited external control. Future studies should further explore how environmental cues, campus safety campaigns, and social influences interact with these MTM components.

This study contributes to the growing body of literature applying the MTM in public health interventions targeting young adults. Compared with previous MTM-based studies on drowsy driving, physical activity, and quitting vaping, the current findings identify behavioral confidence as a consistent determinant of both initiation and sustenance [24,26].

In contrast with other MTM-based research on drowsy driving, physical activity, and quitting smoking e-cigarettes, existing evidence also recognizes confidence in behavior as a consistent determinant of initiation and sustenance [24,25,38]. However, unlike behaviors that occur in fixed settings, texting while driving may require situational triggers and reinforcement strategies tailored to real-time decision making. Thus, digital interventions that constitute push notifications, in-app driving-mode restrictions, or promises of commitments may increase initiation and maintenance outcomes among students.

The results indicate that university-based interventions are necessary to address the psychological and situational factors underlying distracted driving. Interventions can strengthen students’ self-efficacy and emotional regulation skills by practicing behaviors such as behavioral rehearsal, simulation-based training, or mobile phone-use self-monitoring applications [38]. Integration of MTM-informed modules into driver education, campus health-promotion programs, and peer-led workshops may also help normalize non-texting driving behaviors and reinforce sustained behavioral change.

### 4.1. Implications for Practice

The findings of this study offer important insights for designing interventions aimed at reducing texting while driving among college students. With a significant percentage of students reporting engagement in texting while driving within the past month, the need for effective prevention and behavioral change programs is underscored. Addressing this risky behavior requires a multi-level approach that targets both individual and institutional factors. Interventions can be delivered through campus wellness centers, driver safety programs, student affairs offices, or collaborations with motor vehicle departments.

Accessible platforms such as social media, smartphone applications, and peer education initiatives can serve as powerful tools for engaging college students in safe driving campaigns. By meeting students where they already spend their time, these platforms can facilitate real-time interaction, feedback, and motivation to reduce texting while driving.

Applying the Multi-Theory Model (MTM) provides a strong theoretical foundation for understanding both the initiation and maintenance of behavior change related to mobile phone use while driving. Core MTM constructs, including behavioral confidence, emotional transformation, and practice for change, play a pivotal role in shaping students’ decisions and supporting sustained safer driving habits.

Building behavioral confidence can be achieved through practical, skill-based strategies such as encouraging students to enable “Do Not Disturb While Driving” settings, check messages before starting a trip, or use automated responses to minimize distractions. Addressing perceived disadvantages, such as fear of missing urgent communications or social pressure to stay connected, requires awareness campaigns and peer-led discussions that reframe safety as a shared responsibility and a socially valued behavior.

Emotional transformation further deepens engagement by connecting behavior change to personal and moral values. Encouraging students to reflect on the emotional consequences of texting while driving, such as the potential to harm themselves, friends, or others, can foster intrinsic motivation for safer behavior. Positive reinforcement strategies, including sharing testimonials, organizing campus-wide challenges, and offering digital recognition or incentives for consistently safe driving, can help sustain long-term behavior change.

Accessible settings like social networking sites, mobile apps, and peer education courses can serve as powerful tools in encouraging college students to participate in anti-texting-while-driving initiatives. Through contact with students who are already indulging themselves, these settings can enable instant interaction, response, and encouragement of not texting and driving. Using the Multi-Theory Model (MTM) provides a strong theoretical explanation of initiation and maintenance of behavior change regarding mobile phone use and driving.

Emotional change, in turn, increases involvement by connecting behavior change to an individual’s moral personal values. Encouraging students to reflect on the potential harm that texting while driving may cause to themselves, their friends, or others can foster a more profound personal commitment to safe driving behaviors. By implementing positive reinforcement techniques, such as providing digital rewards for consistently driving safely, planning campus-wide competitions, and sharing personal testimonies, behavior change can be sustained, and motivation can be increased. Stress plays a significant role in increasing the likelihood of risky behaviors like texting while driving among college students. By incorporating practices such as Kundalini Yoga, students can learn to manage stress more effectively, improve their focus and impulse control, and ultimately reduce dangerous behaviors such as texting while driving [41,42].

### 4.2. Strengths and Limitations

This research utilized the Multi-Theory Model (MTM), a health behavior’s meta-theory that synthesizes concepts from multiple theories of health behavior and has been proven valuable in public health research [35]. The MTM provides a fresh model of describing the initiation and sustenance phases of behavior change, offering a complete picture of texting while driving among university students. The theoretical strength and empirical foundation of the MTM provide a valid method for measuring cognitive, emotional, and contextual predictors of defensive driving behavior.

One of the strengths of this study includes its theoretical contribution and methodological quality. Applying valid MTM constructs and items with psychometric testing maximized the study’s reliability and internal consistency. Additionally, examining the emerging public health issue of texting and driving adds novelty and importance to the current research base, illustrating the translation of the MTM to a domain beyond the more conventional diet and exercise behavior. The study further advances the MTM’s application for intervening on technology-based risk behavior in young adults.

However, several limitations should be noted. The cross-sectional design of the study limits the ability to draw causal conclusions between MTM constructs and actual behavioral outcomes. Because this study used a cross-sectional design, the SEM analyses assess associations rather than causal or temporal relationships. Therefore, the directionality of the relationships among MTM constructs and refraining from texting while driving cannot be definitively established. The reliance on self-reported data may have introduced biases such as recall bias or social desirability bias, although confidentiality was maintained to minimize these effects. Additionally, the study was conducted among students at a single university in the southwestern United States, which limits its generalizability. Because data were collected from a single Southwestern U.S. university, generalizability is limited. Regional differences in traffic culture, law enforcement intensity, and mobile phone regulations may influence both the prevalence of texting while driving and the cognitive–behavioral predictors associated with refraining from the behavior. States differ in hands-free laws, penalty severity, and enforcement visibility, which may shape behavioral confidence, perceived advantages, and the physical or social environment surrounding TWD. These contextual variations correspond to the distal contextual level described in the contextual mediated model and should be considered when interpreting the findings. Future research should include more diverse student populations to enhance external validity.

Furthermore, the absence of test–retest reliability analysis limits the ability to assess the stability of the constructs over time. Future studies should also consider contextual factors, such as peer influence, driving frequency, and state-level texting laws, to better understand how they might affect texting while driving behavior. The survey did not assess prior crash or near-crash experiences related to texting while driving, which may influence perceptions and behaviors. Future research should incorporate such measures. Because commuting status may influence driving exposure and texting-while-driving behavior, the mixed composition of the sample should be considered when interpreting the findings.

Another limitation is the extremely high correlation between Emotional Transformation and Practice for Change (r = 0.93) in the SEM model, suggesting substantial conceptual overlap between these two constructs. Although each item set was previously subjected to expert face and content validation, it is possible that some items may still be similar in meaning or wording, thereby contributing to multicollinearity and potentially suppressing the significance of the structural paths in the sustenance model. However, because the MTM conceptualizes Emotional Transformation and Practice for Change as theoretically distinct constructs, the current study retained the original specification without combining factors or altering the structure. Future research should carefully re-examine the item content of these two constructs to ensure adequate differentiation and may consider testing alternative model specifications such as evaluating indirect effects or further separating overlapping item content to better clarify their unique and shared contributions within the MTM framework. Despite these limitations, the study provides a strong foundation for future research using the MTM to address texting while driving, offering important insights into the psychosocial and behavioral factors involved.

### 4.3. Recommendations

To effectively reduce texting while driving among college students, prevention strategies should be explicitly aligned with the MTM constructs identified in this study. Strengthening behavioral confidence is essential for helping students initiate behavior change. This can be supported through skills-based training, guided practice, and simulated driving experiences that allow students to rehearse and build confidence in their ability to refrain from texting while driving in real-world conditions. Educational campaigns should also highlight concrete strategies such as enabling “Do Not Disturb While Driving” settings, placing the phone out of reach, or designating a passenger to manage communication, all of which reinforce students’ perceived ability to successfully avoid TWD.

Promoting emotional transformation is equally important for sustaining safer driving habits. Universities and public health organizations can incorporate emotionally engaging elements, such as personal testimonials, immersive driving simulations, and narratives from individuals affected by TWD-related crashes into orientation programs, wellness workshops, and awareness campaigns. These approaches help students reframe their emotional priorities and internal motivations to remain phone-free while driving.

Enhancing practice for change through ongoing self-monitoring tools can support long-term abstinence from TWD. Gamified smartphone applications that track safe driving behaviors and offer rewards or recognition for consistent adherence may reinforce positive habits and encourage sustained behavior change. Universities can further promote these strategies through campus-wide driver safety initiatives and peer-led programs.

Collaborative partnerships among universities, public health departments, and transportation agencies can strengthen the reach and sustainability of these MTM-aligned interventions. Such partnerships can produce coordinated campaigns, community-based simulations, and scalable prevention initiatives tailored to diverse student populations. Future research should employ experimental or longitudinal designs to evaluate the long-term effectiveness of MTM-based interventions and expand sampling across geographically diverse institutions to enhance external validity.

## 5. Conclusions

This study demonstrates that the Multi-Theory Model (MTM) is a practical framework for understanding and predicting both the initiation and sustenance of refraining from texting while driving among college students. The findings highlight that behavioral confidence for initiation and emotional transformation and practice for change for sustenance are the most significant predictors of behavior change, emphasizing the critical role of psychological readiness and emotional regulation in addressing distracted driving.

This study highlights how theory-based interventions can effectively guide real-world strategies to reduce mobile phone use while driving. Programs designed to strengthen drivers’ confidence in their ability to change behavior and to tap into emotional motivation, core elements of the Multi-Theory Model (MTM), show strong potential for promoting safer driving habits. These findings emphasize the importance of building evidence-based, targeted initiatives that not only teach behavioral skills but also support emotional regulation to encourage lasting behavioral change.

Given the relevance of the issue to young adults, the findings of this study call for future interventions to focus on digital tools, behavioral confidence, practice for change, and emotional transformation to sustain long-term changes in texting while driving behavior. Further research should explore the efficacy of MTM-based interventions through longitudinal or experimental designs, examining the long-term impact on behavior change across diverse populations.

## Figures and Tables

**Table 1:** Summary of Demographic Characteristics of the Sample (*n* = 164).

Variable	Characteristics	Mean ± SD	*n* (%)
Age * (*n* = 127)	-	29.9 ± 10.8	-
Working hours per week ** (*n* = 107)	-	31.5 ± 14.1	-
Gender (*n* = 163)	Male	-	45 (72.4)
	Female	-	118 (27.6)
Race and Ethnicity (*n* = 163)	White or Caucasian American	-	61 (37.4)
	Black or African American	-	17 (10.4)
	Asian American	-	29 (17.8)
	American Indian	-	1 (0.61)
	Hispanic American	-	46 (28.2)
	Other	-	9 (5.5)
Class level	Freshmen	-	9 (5.5)
	Sophomore	-	15 (9.2)
	Junior	-	35 (21.3)
	Senior	-	22 (13.4)
	Graduate	-	83 (50.6)
GPA (*n* = 163)	2.00–2.49	-	2 (1.2)
	2.50–2.99	-	12 (7.4)
	3.00–3.49	-	38 (23.3)
	3.50–4.00	-	111 (68.1)
Employment status	Yes	-	107 (84.3)
	No	-	20 (15.8)
Living condition	Off-campus	-	157 (95.7)
	On-campus	-	7 (4.3)
Self-identified problematic texter	Yes	-	47 (28.7)
	No	-	117 (71.3)
Past week’s texting while driving behavior	Never	-	2 (1.2)
	Almost Never	-	60 (36.6)
	Sometimes	-	65 (39.6)
	Fairly Often	-	27 (16.5)
	Often	-	10 (6.1)

* Age data were available for 127 participants. ** Working hours per week were reported by 107 participants.

**Table 2:** Reliability Estimates for the Initiation and Sustenance Scales and Subscales.

Scale	Cronbach’s Alpha (95% CI)
Perceived Advantage	0.81 (0.75, 0.88)
Perceived Disadvantage	0.71 (0.62, 0.79)
Behavioral Confidence	0.93 (0.89, 0.97)
Changes in the Physical Environment	0.86 (0.80, 0.91)
Overall Initiation Scale	0.83 (0.77, 0.90)
Emotional Transformation	0.92 (0.88, 0.97)
Practice for Change	0.88 (0.83, 0.93)
Changes in the Social Environment	0.83 (0.76, 0.89)
Overall Sustenance Scale	0.93 (0.89, 0.97)
Overall Scale	0.93 (0.89, 0.97)

**Table 3:** Descriptive statistics of the multi-theory model of behavior change constructs (*n* = 164).

Constructs	Possible Score Range	Observed Score Range	Mean ± SD
Initiation	0–12	0–12	7.6 ± 3.3
Perceived Advantage (PA)	0–24	0–24	18.8 ± 4.3
Perceived Disadvantage (PDA)	0–24	0–19	7.2 ± 3.8
Participatory Dialogue (PA-PDA)	−24–+24	−16–+23	11.6 ± 6.1
Behavioral confidence	0–24	0–24	16.5 ± 5.8
Changes in the physical environment	0–12	0–12	8.4 ± 3.3
Sustenance	0–12	0–12	7.2 ± 3.3
Emotional transformation	0–12	0–12	7.8 ± 3.2
Practice for change	0–12	0–12	7.3 ± 3.1
Changes in the social environment	0–16	0–16	8.1 ± 4.3

**Table 4:** Hierarchical Multiple Regression Analysis Predicting Initiation Scores.

Variables		Base Model (with Age) *n* = 126		Final Model (with Age) *n* = 126		Base Model (Without Age) *n* = 163		Final Model (Without Age) *n* = 163	
		β	*p*-Value	β	*p*-Value	β	*p*-Value	β	*p*-Value
Intercept		5.26	<0.0001	0.08	0.9409	7.90	<0.0001	1.48	0.0497
Age		0.07	0.0115	0.04	0.0609	-	-	-	-
Sex (Reference Female)		−1.71	0.0091	−1.32	0.0144	−1.92	0.0005	−1.44	0.0007
Self-identified problematic texter (Reference “No”)		0.23	0.7205	0.66	0.2094	0.14	0.8056	0.92	0.0359
Race or Ethnicity (Reference White)	Black	1.63	0.0745	1.65	0.0244	1.22	0.1496	1.37	0.0364
	Asian	1.21	0.1405	1.45	0.0308	0.68	0.3319	0.95	0.0785
	Hispanic	0.77	0.2965	0.23	0.6982	−0.04	0.9503	−0.20	0.6791
	Other Races	−2.89	0.0514	−0.11	0.9272	−2.27	0.0331	−0.73	0.3760
GPA (Reference Higher than 3.5)	Less than 3.0	1.83	0.1410	0.25	0.8048	1.47	0.1333	0.03	0.9657
	Between 3.0 and 3.49	0.60	0.4575	−0.09	0.8925	0.20	0.7632	−0.39	0.4539
Class Level (Reference Graduate)	Freshmen	1.21	0.4497	0.36	0.7820	−0.28	0.8042	−0.32	0.7107
	Sophomore	0.66	0.65	1.12	0.2295	0.64	0.4801	0.82	0.2514
	Junior	0.31	0.7006	−0.09	0.8961	−0.25	0.7201	−0.19	0.7217
	Senior	−0.77	0.4464	−0.94	0.2480	−0.61	0.4540	−0.67	0.2930
Past week’s texting behavior (Reference Somewhat)	Low frequency	0.35	0.5799	0.13	0.8040	1.23	0.0230	0.59	0.1596
	High frequency	−2.03	0.0091	−0.73	0.2616	−1.73	0.0090	−0.75	0.1569
**Participatory Dialogue**		-	-	0.07	0.0859	-	-	0.06	0.0840
**Behavioral Confidence**		-	-	0.27	<0.0001	-	-	0.30	<0.0001
**Changes in the Physical Environment**		-	-	0.08	0.3627	-	-	0.07	0.3629
Adjusted R^2^		0.1742		0.4761		0.1760		0.5151	
ΔR^2^		-		0.3019		-		0.3391	
F		2.76	0.0012	7.31	<0.0001	3.47	<0.0001	11.12	<0.0001

**Table 5:** Hierarchical Multiple Regression Analysis Predicting Sustenance Scores.

Variables		Base Model (with Age) *n* = 126		Final Model (with Age) *n* = 126		Base Model (Without Age) *n* = 163		Final Model (Without Age) *n* = 163	
		β	*p*-Value	β	*p*-Value	β	*p*-Value	β	*p*-Value
Intercept		4.89	<0.0001	0.33	0.7597	7.44	<0.0001	1.48	0.0185
Age		0.07	0.0073	0.05	0.0388	-	-	-	-
Sex (Reference Female)		−2.20	0.0004	−1.61	0.0021	−2.14	<0.0001	−1.43	0.0003
Self-identified problematic texter (Reference “No”)		1.45	0.0197	1.68	0.0012	1.09	0.0478	1.63	<0.0001
Race or Ethnicity (Reference White)	Black	1.69	0.0495	1.66	0.0189	1.15	0.1668	0.51	0.3904
	Asian	0.29	0.7048	0.32	0.6191	−0.13	0.8511	−0.33	0.5017
	Hispanic	0.28	0.6922	−0.39	0.5003	−0.53	0.3767	−0.31	0.4702
	Other Races	−2.59	0.0628	−0.29	0.8036	−2.26	0.0304	−0.87	0.2485
GPA (Reference Higher than 3.5)	Less than 3.0	1.48	0.2052	0.24	0.8083	1.87	0.0528	0.89	0.2050
	Between 3.0 and 3.49	0.28	0.7093	−0.44	0.4823	0.11	0.8645	0.07	0.8825
Class Level (Reference Graduate)	Freshmen	1.99	0.1867	1.03	0.4071	−0.08	0.9406	−0.17	0.8327
	Sophomore	0.81	0.4511	1.08	0.2279	1.01	0.2577	1.46	0.0245
	Junior	1.09	0.1565	0.55	0.3866	0.32	0.6347	0.30	0.5294
	Senior	−0.51	0.5923	−0.38	0.6239	−0.49	0.5453	−0.12	0.8331
Past week texting behavior (Reference Somewhat)	Low frequency	0.33	0.5751	0.16	0.7448	1.20	0.0240	0.71	0.0607
	High frequency	−2.90	<0.0001	−1.67	0.0083	−2.23	0.0007	−1.00	0.0357
**Emotional Transformation**		-	-	0.13	0.0020	-	-	0.41	<0.0001
**Practice for change**		-	-	0.15	0.0083	-	-	0.27	0.0105
**Changes in the Social Environment**		-	-	0.19	0.0360	-	-	0.05	0.3447
Adjusted R^2^		0.2834		0.5227		0.2417		0.6147	
ΔR^2^		-		0.2393		-		0.3730	
F		4.29	<0.0001	8.60	<0.0001	4.69	<0.0001	16.20	<0.0001

## Data Availability

Due to ethical restrictions, the data obtained and analyzed during the study are not publicly available and can be obtained from the corresponding author upon reasonable request.

## Supplementary Materials

The following supporting information can be downloaded at: https://www.mdpi.com/article/10.3390/ijerph22121847/s1, File S1: Survey Questionnaire.

## Abbreviations

The following abbreviations are used in this manuscript:

TWD	Texting while driving
TPB	Theory of Planned Behavior
HBM	Health Belief Model
PWM	Prototype Willingness Model
MTM	Multi-Theory Model
SEM	Structural equation modeling
CFI	Comparative Fit Index
TLI	Tucker–Lewis Index
RMSEA	Root Mean Square Error of Approximation
SRMR	Standardized Root Mean Square Residual
VIF	Variance Inflation Factor
