# Supplementary material for: Theory-Based Antecedents of Stopping Texting While Driving Among College Students for Injury Prevention: A Cross-Sectional Study
Source: Int J Environ Res Public Health. 2025 Dec 10;22(12):1847. doi: 10.3390/ijerph22121847 (PMC12732351; doi:10.3390/ijerph22121847)
Supplement: Supplementary file 1 [file ijerph-22-01847-s001.zip › ijerph-3963383-supplementary.pdf]

MEASURING CHANGE IN STOPPING TEXTING WHILE DRIVING BEHAVIOR

Directions: Please answer the following questions to the best of your abilities.

1. Do you have a cell phone?

   - ☐ Yes
   - ☐ No

If you marked no, please stop taking the survey. Thank you for your time!

…………………………………………………………………………………………………….

2. Do you drive?

   - ☐ Yes
   - ☐ No

If you marked no, please stop taking the survey. Thank you for your time!

…………………………………………………………………………………………………….

3. Have you texted while driving in the past 30 days?

   - ☐ Yes
   - ☐ No
   - ☐ Not sure

…………………………………………………………………………………………………….

4. During the past week how often did you text while driving?

   Never Almost Never Sometimes Fairly Often Often

…………………………………………………………………………………………………….

	Never	Almost Never	Sometimes	Fairly Often	Very Often
5. If you stop texting while driving, you will not get into an accident.					
6. If you stop texting while driving, you can develop the habit of staying focused on the road, ensuring safer driving practices.					
7. If you stop texting while driving, you will not be distracted while driving.					
8. If you stop texting while driving, you can enjoy greater peace of mind, knowing you are driving safely.					
9. If you stop texting while driving, your family will feel more at ease, knowing you prioritize your safety on the road.					
10. If you stop texting while driving, you will avoid traffic violations/tickets.					

	Never	Almost Never	Sometimes	Fairly Often	Very Often
11. If you stop texting while driving, you cannot respond to text messages on time.					
12. If you stop texting while driving, you will feel guilty.					
13. If you stop texting while driving, you will miss opportunities.					
14. If you stop texting while driving, you will not get immediate gratification.					
15. If you stop texting while driving, you will forget important things.					
16. If you stop texting while driving, you may lose friendships.					

	Not At All Sure	Slightly Sure	Moderately Sure	Very Sure	Completely Sure
17. How sure are you that you can stop texting while driving this week?					
18. How sure are you that you can stop texting while driving this week even if you are expecting an important text message?					
19. How sure are you that you can stop texting while driving this week even if you must multitask?					
20. How sure are you that you can stop texting while driving this week even if you want to communicate with friends?					
21. How sure are you that you can stop texting while driving this week even if you want to communicate with family?					
22. How sure are you that you can set a goal to stop texting while driving?					

	Not At All Sure	Slightly Sure	Moderately Sure	Very Sure	Completely Sure
23. How sure are you that you can stop texting while driving this week by removing the notification beep for text messages while driving?					
24. How sure are you that you can stop texting while driving this week by putting away your cell phone while driving?					

25. How sure are you that you can stop texting while driving this week by removing all signals from your environment to respond to text messages while driving?

……………………………………………………………………………………………………

Not At All Sure	Slightly Sure	Moderately Sure	Very Sure	Completely Sure

26. How sure are you that you can direct your feelings to the goal of stopping texting while driving from now on?

……………………………………………………………………………………………………

27. How sure are you that you can motivate yourself to stop texting while driving from now on?

……………………………………………………………………………………………………

28. How sure are you that you can overcome self-doubt in accomplishing the goal of stopping texting while driving from now on?

……………………………………………………………………………………………………

Not At Sure	Slightly Sure	Moderately Sure	Very Sure	Completely All Sure

29. How sure are you that you can monitor your goal of stopping texting while driving from now on?

……………………………………………………………………………………………………

30. How sure are you that you can stop texting while driving from now on if you encounter barriers (e.g., inconvenience, time pressure, etc.)?

……………………………………………………………………………………………………

31. How sure are you that you can adapt to stopping texting while driving from now on?

……………………………………………………………………………………………………

Not At All Sure	Slightly Sure	Moderately Sure	Very Sure	Completely Sure

32. How sure are you that a family member can help you stop texting while driving from now on?

……………………………………………………………………………………………………

33. How sure are you that a friend can help you to stop texting while driving from now on?

……………………………………………………………………………………………………

34. How sure are you that social media can help you to stop texting while driving from now on?

……………………………………………………………………………………………………

35. How sure are you that you can take the help of a passenger driving with you to avoid texting while driving?

……………………………………………………………………………………………………

Not At All Likely	Somewhat Likely	Moderately Likely	Very Likely	Completely Likely

36. How likely is it that you will stop texting while driving in the next week?

…………………………………………………………………………………………………..

37. How likely do you want to stop texting while driving in the next week?

……………………………………………………………………………………………………

38. How likely do you intend to stop texting while driving in the next week?

…………………………………………………………………………………………………

39. How likely is it that you will stop texting while driving from now on?

…………………………………………………………………………………………………

40. How likely do you want to stop texting while driving from now on?

…………………………………………………………………………………………………

41. How likely do you intend to stop texting while driving from now on?

…………………………………………………………………………………………………

42. Do you consider texting while driving to be a problem for you?

- ☐ Yes
- ☐ No

…………………………………………………………………………………………………

43. What is your gender?

- ☐ Male
- ☐ Female
- ☐ Other, ________________

…………………………………………………………………………………………………

44. How old are you today? _______ years

…………………………………………………………………………………………………

45. What is your race/ethnicity?

- ☐ White or Caucasian American
- ☐ Black or African American
- ☐ Asian American
- ☐ American Indian
- ☐ Hispanic American
- ☐ Other _________________

…………………………………………………………………………………………………

46. What is your class?

- ☐ Freshmen
- ☐ Sophomore
- ☐ Junior
- ☐ Senior
- ☐ Graduate

…………………………………………………………………………………………………

47. What is your current overall GPA? (on a 4.00 scale)

- ☐ Less than 1.99
- ☐ 2.00 – 2.49
- ☐ 2.50 – 2.99
- ☐ 3.00 – 3.49
- ☐ 3.50 – 4.00

…………………………………………………………………………………………………

48. Where do you live?

- ☐ On campus
- ☐ Off-campus

…………………………………………………………………………………………………

49. Do you work?

- ☐ No

□ Yes, _____ average hours per week (put a single number, not a range)

……………………………………………………………………………………………………

Thank you for your time!

SCORING

The construct of advantages: Scale: Never (0), Almost never (1), Sometimes (2), Fairly often (3), Very often (4). The summative score of Items 5-10. Possible range: 0- 24 units. A high score is associated with the likelihood of initiation of behavior change.

The construct of disadvantages: Scale: Never (0), Almost never (1), Sometimes (2), Fairly often (3), Very often (4). The summative score of Items 11-16. Possible range: 0- 24 units. A low score is associated with the likelihood of initiation of behavior change.

Subtract the disadvantages score from the advantages score to calculate the **participatory dialogue** construct score. Possible range: -24 to +24 units. A positive score will be indicative of behavior change.

The construct of behavioral confidence: Scale: Not at all sure (0), slightly sure (1), moderately sure (2), very sure (3), completely sure (4). The summative score of Items 17-22. Possible range 0-24 units. A high score is associated with the likelihood of initiation of behavior change.

The construct of changes in the physical environment: Scale: Not at all sure (0), slightly sure (1), moderately sure (2), very sure (3), completely sure (4). The summative score of Items 23-25. Possible range 0-12 units. A high score is associated with the likelihood of initiation of behavior change.

The construct of emotional transformation: Scale: Not at all sure (0), slightly sure (1), moderately sure (2), very sure (3), completely sure (4). The summative score of Items 26-28. Possible range 0-12. A high score is associated with the likelihood of sustenance of behavior change.

The construct of practice for change: Scale: Not at all sure (0), slightly sure (1), moderately sure (2), very sure (3), completely sure (4). The summative score of Items 29-31. Possible range 0-12. A high score is associated with the likelihood of sustenance of behavior change.

The construct of changes in the social environment: Scale: Not at all sure (0), slightly sure (1), moderately sure (2), very sure (3), completely sure (4). The summative score of Items 32-35. Possible range 0-16. A high score is associated with the likelihood of sustenance of behavior change.

The construct of intent to initiate: Scale: Not at all likely (0), somewhat likely (1), moderately likely (2), very likely (3), and completely likely (4). The summative score of Items 36-38. Possible range 0-12.

The construct of intent to sustain: Scale: Not at all likely (0), somewhat likely (1), moderately likely (2), very likely (3), and completely likely (4). The summative score of Items 39-41. Possible range 0-12.

Flesch Reading Ease: 79.7
Flesch-Kincaid Grade Level: 5.4

© Manoj Sharma
